# Combined Body Mass Index and Body Surface Area to Predict Post Kidney Transplant Outcomes in Patients With Obesity

**DOI:** 10.1097/TXD.0000000000001807

**Published:** 2025-05-21

**Authors:** Roxaneh Zaminpeyma, Louise Moist, Kristin K. Clemens, Michael Chiu, Janet Madill, Karthik Tennankore, Amanda J. Vinson

**Affiliations:** 1 Faculty of Medicine, Dalhousie University, Halifax, NS, Canada.; 2 Division of Nephrology, Department of Medicine, Schulich School of Medicine and Dentistry, Western University, London, ON, Canada.; 3 Institute for Clinical Evaluative Sciences, London, ON, Canada.; 4 Division of Endocrinology and Metabolism, Department of Medicine, Western University, London, ON, Canada.; 5 Lawson Research Institute, London, ON, Canada.; 6 Department of Epidemiology and Biostatistics, Western University, London, ON, Canada.; 7 Brescia School of Food and Nutritional Sciences, Western University, London, ON, Canada.; 8 Division of Nephrology, Department of Medicine, Dalhousie University, Halifax, NS, Canada.; 9 Kidney Research Institute Nova Scotia (KRINS), Halifax, NS, Canada.

## Abstract

**Background.:**

The prevalence of obesity is increasing in both the general and kidney failure populations. Severe obesity (body mass index [BMI] ≥ 40 kg/m^2^) is considered by many centers to be a barrier to kidney transplantation (KT). Obesity is typically defined using BMI. Body surface area (BSA) is not considered, though may also be important.

**Methods.:**

We examined post-KT adverse outcomes associated with obesity defined using combined BMI-BSA parameters in a cohort of adult KT recipients (living/deceased donor) across the United States (Scientific Registry of Transplant Recipients: 2000–2017). Recipient obesity was defined as BMI ≥30 kg/m^2^, or BSA ≥1.94 m^2^ in women and ≥2.17 m^2^ in men. We used multivariable cox proportional hazards or logistic regression models as appropriate to assess the association between BMI-BSA-defined obesity with death-censored graft loss, all-cause graft loss, and delayed graft function.

**Results.:**

The final study included 242 432 patients; 77 556 (32.0%) had obesity based on BMI and 67 312 (28.6%) had obesity based on BSA. Compared to patients with a nonobese BMI and BSA, the adjusted risk of death-censored graft loss, all-cause graft loss, and delayed graft function was greatest when both BMI and BSA indicated obesity (adjusted hazard ratio 1.23, 95% confidence interval [CI]: 1.20-1.27, adjusted hazard ratio 1.09, 95% CI: 1.07-1.11, adjusted odds ratio 1.58, 95% CI: 1.53-1.63, respectively); a significantly greater risk than when BMI and BSA were discordant.

**Conclusions.:**

Currently only BMI is considered when evaluating obesity-related KT risk; however, combined BMI-BSA obesity may better identify individuals at high risk of poor outcomes posttransplant than BMI alone.

## Graphical abstract

**Figure:**
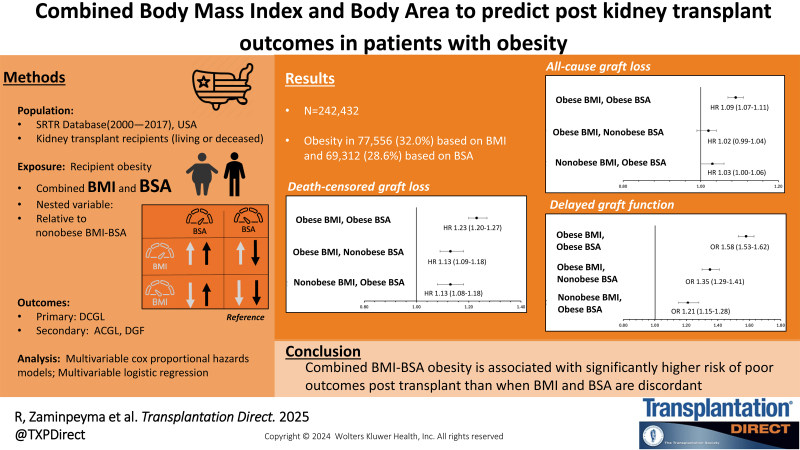

Obesity is associated with the development of kidney disease,^1^ with an increasing prevalence of obesity in the general and kidney failure populations alike.^2,3^ Kidney transplantation (KT) is the optimal treatment for eligible patients with kidney failure.^4^ Despite this, access is limited for patients with severe obesity, in part, because of the varying risks associated with obesity and adverse peri- and posttransplantation outcomes.^5-9^ These outcomes include a greater risk of delayed graft function (DGF),^10-13^ graft loss, and rejection.^6,7,14,15^ Obesity is also associated with increased risk of infection, poor wound healing, and serious complications such as fascial dehiscence which may require reintervention.^5,16^ Because transplantation is associated with a survival advantage compared with those remaining on dialysis (even in patients living with obesity),^17^ parameters that are used to assess obesity need to be evaluated closely.

Obesity is generally defined as a body mass index (BMI) ≥ 30 kg/m^2^; other body composition parameters such as body surface area (BSA) are not typically considered but may also be important. Although BMI looks at body fat composition, BSA estimates metabolic mass^18^; BSA thresholds to identify obesity have been suggested but are not been clearly established.^19^ BSA’s measure of weight and height has been widely used for dose calculation of chemotherapies and evaluation of cardiac function.^20,21^ In transplantation literature, BSA has been used to assess donor-recipient size mismatch.^22^ Studies have correlated BSA with renal parenchymal volume, suggesting potential association with renal function^23^; contemporary eGFR equations ubiquitously standardize renal function for 1.73 m^2^ BSA.^24-26^

To our knowledge, BSA-defined obesity has not been studied as a clinical predictor of risk after KT. Therefore, the purpose of this study was to examine the risk of short- and long-term outcomes after KT in patients with obesity defined using (1) BMI, (2) BSA, and (3) combined BMI-BSA, relative to those without obesity.

## MATERIALS AND METHODS

### Subject Selection

We conducted a retrospective cohort study of adult KT recipients, who received a kidney from either a living or deceased donor in the United States from 2000 to 2017, using the Scientific Registry of Transplant Recipients (SRTR). We excluded patients <18 y of age, those who underwent multiple, en bloc, or sequential transplants, those with missing values for BMI or BSA, and those with a reported BMI <15 or >100 kg/m^2^ or BSA <1.0 or >3.2 m^2^ because these were felt to represent coding errors. The study protocol was submitted to the Nova Scotia Health Research Ethics Board who deemed the study met requirements outlined in the Tri-Council Policy Statement for an REB review exemption (REB FILE no.: 1028869).

### Exposure

The primary exposure was recipient obesity defined using both BMI and BSA. Obesity was defined as BMI ≥30 kg/m^2^ as per the widely accepted World Health Organization definition (weight/[height]^2^).^27^ There are no established or validated thresholds to define obesity based on BSA; therefore, for the purposes of this study, the BSA threshold for obesity was determined to be one standard deviation above the mean BSA for overweight (BMI 25–29.9 kg/m^2^) men and women separately, as demonstrated in earlier literature. This resulted in a BSA threshold of ≥1.94 m^2^ in women and ≥2.17 m^2^ in men.^19^ BSA was calculated using Mosteller’s equation ([recipient height × recipient weight]/[3600]^½^) because the Dubois-Dubois equation has been shown to underestimate BSA in patients with obesity.^19,20,28^ Nonobese BMI was defined as <30 kg/m^2 27^ and nonobese BSA as <1.94 m^2^ (women) or <2.17 m^2^ (men).^19^ For our primary analysis, we created a nested variable of combined BMI-BSA obesity:

1. Nonobese BMI-nonobese BSA (reference category);
2. Obese BMI-nonobese BSA;
3. Nonobese BMI-obese BSA;
4. Obese BMI-obese BSA.

In secondary analyses, we separately examined the association of BMI-defined obesity relative to nonobese BMI, and BSA-defined obesity relative to nonobese BSA (as defined above) with the risk of each primary and secondary outcome.

### Outcomes

The primary outcome was death-censored graft loss (DCGL), defined as return to dialysis or preemptive retransplantation. Secondary outcomes included all-cause graft loss (ACGL), DGF (defined as the need for dialysis in the first week posttransplant), and patient survival.

### Statistical Analysis

Baseline characteristics were presented for patients using counts (percentage) or medians (interquartile ranges) where appropriate.

We performed univariable and multivariable cox proportional hazards models to assess the risk of combined BMI-BSA-defined obesity with DCGL and ACGL, and multivariable logistic regression to assess the odds of BMI-BSA-defined obesity with DGF. Regression analyses were adjusted for preemptive KT status, previous KT, cause of kidney failure, donor and recipient age, race, sex, donor BMI, panel-reactive antibodies, HLA mismatch, donor type (living versus deceased), and recipient comorbidities including diabetes mellitus, peripheral vascular disease, coronary artery disease, and hypertension. Complete case analysis was used for all analyses. Time to DCGL and ACGL by BMI-BSA classification was demonstrated using Kaplan–Meier survival curves.

In secondary analyses, we *separately* examined the independent association of recipient obesity defined using literature thresholds for either BMI or BSA (not combined BMI-BSA) with each of DCGL, ACGL, and DGF using multivariable Cox or logistic regression models as outlined earlier.

We created multivariable regression models that included BMI, BSA and an interaction term between the 2 to determine if BSA modified the known association between BMI and each primary and secondary outcome.

In sensitivity analyses, we used restricted cubic splines to categorize our continuous predictors (BMI and BSA) as described previously,^29^ rather than based on existing thresholds/suggested thresholds. Cubic splines produce a series of knots, or end points, which best approximate a function and are felt to more optimally categorizes continuous data. Because it is believed that sex may influence BSA thresholds for obesity, in this data driven model, we looked at knots for both BMI and BSA after stratifying by sex, and identified the terminal knot for each variable (of 3) to use as our threshold for “obesity” or risk. We then used these new thresholds for BMI and BSA to create a new nested data-driven BMI-BSA variable and repeated our primary analyses with this new nested variable. Finally, our primary analyses were repeated using the DuBois formula, BSA = weight^0.425^ × height^0.725^ × 0.007184^30^ to define patient obesity (with thresholds defined as earlier; ≥2.15 m^2^ for men and ≥1.91 m^2^ for women).^19^

This study used data from the SRTR. The SRTR data system includes data on all donor, wait-listed candidates, and transplant recipients in the United States, submitted by the members of the Organ Procurement and Transplantation Network. The Health Resources and Services Administration, US Department of Health and Human Services, provides oversight to the activities of the Organ Procurement and Transplantation Network and SRTR contractors. The data reported here have been supplied by the Hennepin Healthcare Research Institute as the contractor for the SRTR. The interpretation and reporting of these data are the responsibility of the author(s) and in no way should be seen as an official policy of or interpretation by the SRTR or the US Government. All statistical analyses were performed using Stata version 13.1 (Stata Corp., College Station, TX). For statistical comparisons, a *P* < 0.05 was deemed the threshold for statistical significance.

## RESULTS

**Figure 1.**
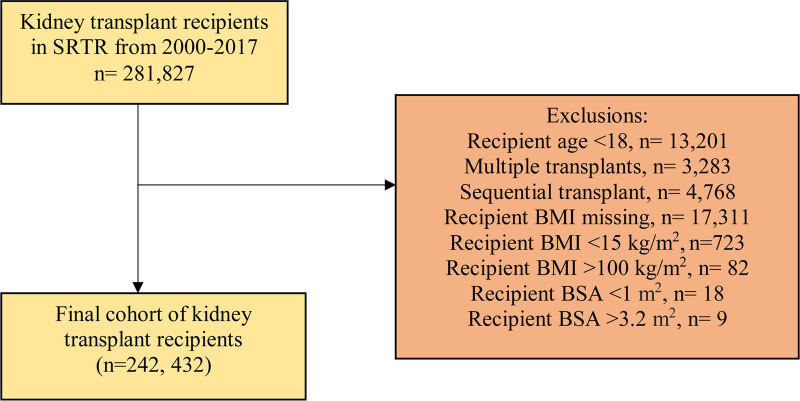
Kidney transplant cohort derivation following exclusions in BMI-BSA study. BMI, body mass index; BSA, body surface area; SRTR, Scientific Registry of Transplant Recipients.

After relevant exclusions, a total of 242 432 patients were included in the study, Figure 1. Baseline characteristics stratified by BMI-BSA pairing status are shown in Table 1. Overall, 77 556 (32.0%) patients had obesity based on BMI thresholds and 69 312 (28.6%) had obesity based on BSA thresholds. 53 634 (22.1%) patients had concordance for obese BMI and BSA and 149 198 (61.5%) patients had concordance for nonobese BMI and BSA (83.6% concordance overall). Discordance was observed in 16.4% of patients: 15 678 (6.5%) patients had nonobese BMI and obese BSA, and 23 922 (9.9%) patients had obese BMI and nonobese BSA.

**TABLE 1.** Baseline characteristics of study cohort at kidney transplantation and post–kidney transplantation

Nested BMI-BSA variables, N = 242 432	Nonobese BMI-nonobese BSA, N = 149 198 (61.5%)	Nonobese BMI-obese BSA, N = 15 678 (6.5%)	Obese BMI-nonobese BSA, N = 23 922 (9.9%)	Obese BMI-obese BSA, N = 53 634 (22.1%)
Age, y, median (IQR)	51 (39-61)	52 (43-60)	55 (44-63)	52 (43-60)
Sex (male), n (%)	89 440 (60.0)	11 602 (74.0)	13 513 (56.5)	32 865 (61.3)
Race, n (%)				
White	102 515 (68.7)	10 227 (65.2)	16 777 (70.1)	34 805 (64.9)
Black	33 108 (22.1)	5177 (33.0)	5479 (22.9)	17 282 (32.2)
Other	13 575 (9.1)	274 (1.8)	1664 (7.0)	1546 (2.9)
Comorbidities, n (%)				
Diabetes	37 995 (25.7)	4808 (30.9)	10 294 (43.4)	23 094 (43.4)
Hypertension	113 489 (87.3)	12 084 (89.0)	18 559 (89.2)	41 563 (89.8)
Peripheral vascular disease	6720 (4.7)	858 (5.7)	1541(6.7)	3611 (6.9)
Coronary artery disease	9762 (8.0)	1124 (8.9)	2024 (10.6)	4213 (10)
Kidney failure diagnosis, n (%)				
Diabetes	29 760 (20.9)	3686 (24.1)	8262 (35.8)	17 781 (34.0)
Polycystic kidney disease	14 036 (9.9)	2615 (17.1)	1412 (6.1)	5110 (9.8)
Glomerulonephritis	39 383 (27.7)	3625 (23.8)	4802 (20.8)	11 752 (22.5)
Hypertension	34 571 (24.3)	3573 (23.4)	5772 (25.0)	12 494 (23.9)
Other	24 490 (17.2)	1776 (11.6)	2837 (12.3)	5,193 (9.9)
Kidney transplant history, n (%)				
Preemptive kidney transplant	27 946 (18.8)	3358 (21.5)	3985 (16.7)	9550 (17.9)
Previous kidney transplant	21 354 (14.3)	1652 (10.5)	2278 (9.5)	4,226 (7.9)
Panel-reactive activity, n (%)				
0	98 471 (82.0)	10 855 (85.5)	15 287 (81.7)	35 321 (83.9)
1	14 905 (12.4)	1321 (10.4)	2454 (13.1)	4934 (11.7)
2	6646 (5.5)	516 (4.1)	981 (5.2)	1871 (4.4)
HLA mismatch, n (%)				
0	13 373 (9.0)	1212 (7.8)	1970 (8.3)	4238 (8.0)
1	5602 (3.8)	484 (3.1)	794 (3.3)	1715 (3.2)
2	13 650 (9.2)	1329 (8.5)	1887 (7.9)	4375 (8.2)
3	27 250 (18.4)	2805 (18.0)	4226 (17.8)	9473 (17.8)
4	31 740 (21.4)	3523 (22.6)	5386 (22.7)	12 089 (22.7)
5	37 256 (25.2)	4138 (26.6)	6207 (26.1)	14 219 (26.7)
6	19 230 (13.0)	2094 (13.4)	3303 (13.9)	7156 (13.4)
Donor type, n (%)				
Living	56 077 (37.6)	6080 (38.8)	7625 (31.9)	18 968 (35.4)
Deceased	93 121 (62.4)	9598 (61.2)	16 297 (68.1)	34 666 (64.6)
Donor BMI ≥30 kg/m^2^, n (%)	34 857 (23.8)	4006 (26.0)	6655 (28.2)	15 875 (30.0)

Missing data: race: N = 3 (<1%); diabetes: N = 2311 (<1%); hypertension: 30 794 (13%); peripheral vascular disease: 8154 (3%); CAD: 46 187 (19%); kidney failure diagnosis: 9502 (4%); preemptive transplant: 1290 (<1%); panel-reactive activity: 48 870; HLA mismatch: 1708 (<1%); donor BMI: 4135 (2%).

BMI, body mass index; BSA, body surface area; IQR, interquartile range.

The median age was 53 y and 147 420 (60.8%) were male; age was similar across BMI-BSA categories, and male sex ranged from 56.5% (obese BMI-nonobese BSA) to 74.0% (nonobese BMI-obese BSA). Hypertension was present in 87.7%, and diabetes in 31.7% of recipients. As expected in BMI-defined obesity, the prevalence of diabetes was higher in the obese BMI-BSA (43.4%) and the obese BMI-nonobese BSA (43.4%) cohorts. The prevalence of other comorbidities appeared otherwise similar across BMI-BSA pairings. Glomerulonephritis (20.8%–27.7%) was the most common primary diagnosis of kidney failure in the nonobese BMI-BSA. Diabetes (34.0%–35.8%) was the most common primary diagnosis among obese BMI (irrespective of BSA status). Overall, 153 682 (63.4%) patients received a kidney from a deceased donor; patients with an obese BMI-nonobese BSA were slightly less likely to receive a living donor (31.9%) compared with patients with a nonobese BMI (37.6%–38.8%). Additionally, the donor was more likely to have a BMI ≥30 kg/m^2^ when the recipient had combined obese BMI-BSA (30.0%) compared with when the recipient had nonobese BMI-BSA (23.8%).

**Figure 2.**
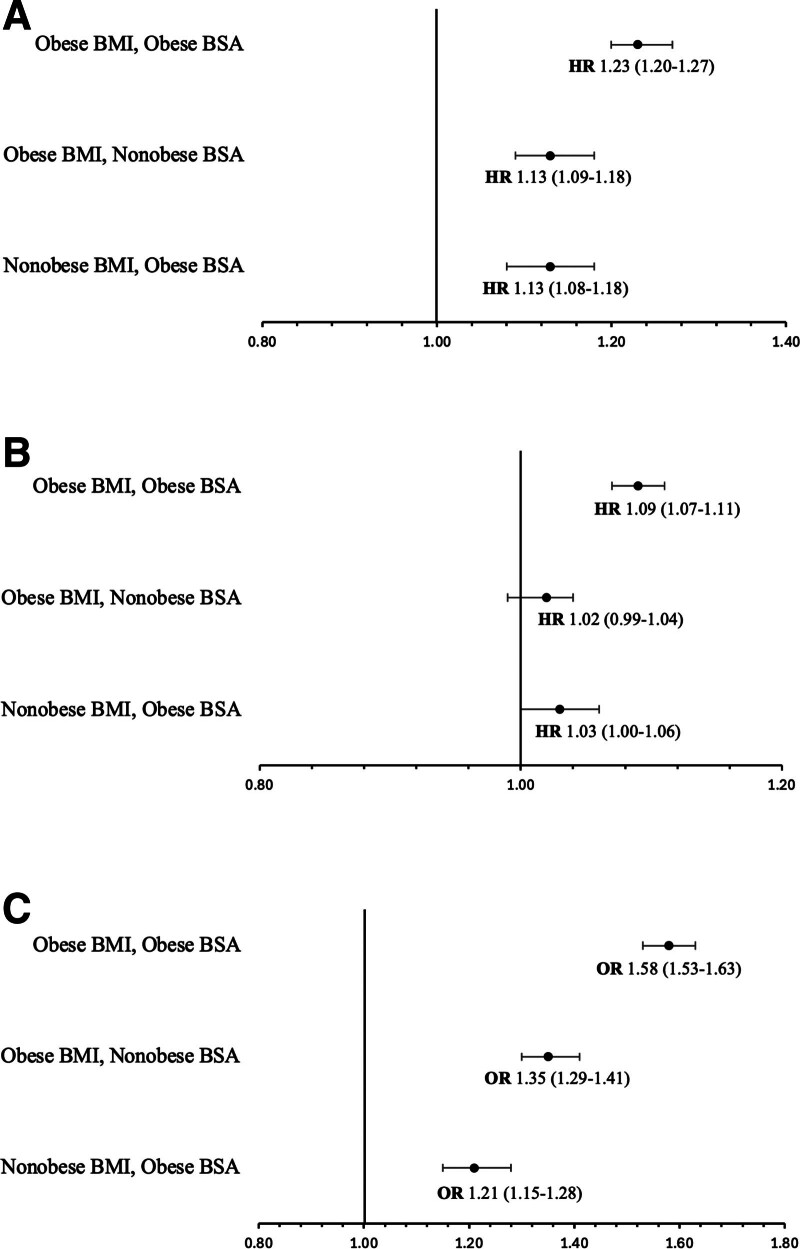
Death-censored graft loss, all-cause graft loss, delayed graft function risk based on BMI-BSA pairing for transplant recipients with obesity. A, Death-censored graft loss based on BMI-BSA pairing for transplant recipients with obesity. B, All-cause graft loss based on BMI-BSA pairing for transplant recipients with obesity. C, Delayed graft function risk based on BMI-BSA pairing for transplant recipients with obesity. Adjusted for preemptive kidney transplant, previous kidney transplant, cause of end-stage kidney disease, donor and recipient age, race, sex, donor body mass index, panel-reactive antibody, HLA mismatch, donor type, and recipient comorbidities (diabetes, peripheral vascular disease, coronary artery disease, hypertension). BMI, body mass index; BSA, body surface area; HR, hazard ratio; OR, odds ratio.

DCGL occurred in 32 718 (13.5%) and ACGL in 68 513 (28.3%); median follow-up time was 4.23 (Q1 1.98, Q3 7.79) y. In our primary analysis, relative to patients with nonobese BMI-BSA, the adjusted risk of DCGL was highest in the setting of combined elevated BMI-BSA (adjusted hazard ratio [aHR] 1.23, 95% confidence interval [CI]: 1.20-1.27), followed by obese BMI-nonobese BSA (aHR 1.13, 95% CI: 1.09-1.18) and nonobese BMI-obese BSA (aHR 1.13, 95% CI: 1.08-1.18) (Figure 2A). For the outcomes of ACGL and DGF, relative to nonobese BMI-BSA, an obese BMI-BSA was highest risk for both (aHR 1.09, 95% CI: 1.07-1.11 for ACGL [Figure 2B]; aOR 1.58, 95% CI: 1.53-1.63 for DGF [Figure 2C]). Importantly, for each outcome (DCGL, ACGL, and DGF) in addition to being higher risk than nonobese BMI-BSA, obese BMI-BSA was also associated with a higher risk than when BMI and BSA were discordant (obese BMI-nonobese BSA or nonobese BMI-obese BSA) (Figure 2A-C). The differences between the risk of obese BMI-BSA, obese BMI-nonobese BSA, and nonobese BMI-obese BSA were each significant for the outcome of DGF, with obese BMI-BSA having the highest risk followed by obese BMI-nonobese BSA (Figure 2C). Kaplan–Meier survival curves examining time to DCGL and ACGL are depicted in **Figure S1** (**SDC**, https://links.lww.com/TXD/A763) (*P* < 0.001 for each). BMI-BSA did not associate with patient survival other than a small but significant reduced mortality in patients categorized as obese by BMI but nonobese by BSA criteria (**Figure S2, SDC,** https://links.lww.com/TXD/A763).

In secondary analyses, obesity defined by BMI alone was associated with risk of DCGL (aHR 1.19, 95% CI: 1.16-1.21), ACGL (aHR 1.06, 95% CI: 1.04-1.08), and DGF (aOR 1.47, 95% CI: 1.43-1.51) relative to nonobese BMI (Table 2). BSA-defined obesity was very similarly associated with DCGL (aHR 1.19, 95% CI: 1.16-1.22), ACGL (aHR 1.07, 95% CI: 1.05-1.09), and DGF (aOR 1.42, 95% CI: 1.38-1.46) relative to nonobese BSA. Obese BSA did not modify the risk of elevated BMI for the outcomes of DCGL (*P* = 0.227), ACGL (*P* = 0.089), or DGF (*P* = 0.264).

**TABLE 2.** DCGL, ACGL, and DGF outcomes by recipient obesity definitions

	DCGL (HR)	ACGL (HR)	DGF (OR)
Obese BMI-obese BSA	1.23 (1.20–1.27)	1.09 (1.07–1.11)	1.58 (1.53–1.63)
Obese BMI	1.19 (1.16–1.21)	1.06 (1.04–1.08)	1.47 (1.43–1.51)
Obese BSA	1.19 (1.16–1.22)	1.07 (1.05–1.09)	1.42 (1.38–1.46)
BMI and BSA interaction	*P* = 0.227	*P* = 0.089	*P* = 0.264

All models were adjusted for preemptive kidney transplant, previous kidney transplant, cause of end-stage kidney disease, donor and recipient age, race, sex, donor body mass index, panel reactive antibody, HLA mismatch, donor type, and recipient comorbidities (diabetes, peripheral vascular disease, coronary artery disease, hypertension).

ACGL, all-cause graft loss; BMI, body mass index; BSA, body surface area; DGCL, death-censored graft loss; DGF, delayed graft function; HR, hazard ratio; OR, odds ratio.

Univariable analyses examining the association between combined BMI-BSA obesity, BMI-defined obesity, and BSA-defined obesity with each outcome separately were similar, and are depicted in **Table S1** (**SDC**, https://links.lww.com/TXD/A763). Finally, when using cubic splines to categorize BMI and BSA instead of existing thredholds, the sex-specific thresholds used for BMI “obesity” were 34.8 kg/m^2^ for men and 35.8 kg/m^2^ for women and for BSA were 2.37 m^2^ for men and 2.17 m^2^ for women. With these new thresholds to create a data-driven BMI-BSA variable, results were overall similar, acknowledging wider CIs with a smaller proportion categorized as obese based on both BMI and BSA (**Table S2, SDC,** https://links.lww.com/TXD/A763). Finally, defining BSA thresholds for obesity using the DuBois formula yielded nearly identical results as when the Monsteller formula was used (**Table S3, SDC,** https://links.lww.com/TXD/A763).

## DISCUSSION

In this study, we show that when both BMI and BSA parameters were consistent with definitions of obesity (obese BMI-BSA), the risk of DCGL, ACGL and DGF were all greater than when neither BMI nor BSA met threshold for obesity (nonobese BMI-BSA). The combination of an obese BMI-BSA was also associated with a significantly higher risk for each outcome than when there was discordance between BMI and BSA (one elevated but not the other). In other words, patients with discordance between obesity-defined BMI and BSA are at significantly lower risk than those categorized as obese by both BMI and BSA metrics.

BMI-BSA-defined obesity is a novel metric which has not been studied in KT to date. BMI is commonly used to define obesity-related thresholds for transplant eligibility, whereas other anthropometric measures of obesity are rarely considered—though may be of importance. For example, in our study, recipients with an obese BMI but nonobese BSA were at increased risk for DCGL (aHR 1.13, 95% CI: 1.09-1.18); however, that risk was equivalent among patients with a *nonobese* BMI but obese BSA (aHR 1.13, 95% CI: 1.08-1.18). This suggests that the use of BMI alone could potentially misclassify patients with obesity-related transplant risk. Additionally, given existing literature,^31-33^ we had expected patients with obese BMI to be at risk for ACGL irrespective of their BSA, however interestingly, patients with an obese BMI but nonobese BSA were not at an expected increased risk (aHR 1.02, 95% CI: 0.99-1.04). Although ours is the first study to examine the influence of BSA in evaluating obesity-related kidney transplant risk, it has been suggested in the general population that when BSA is combined with vertical trunk circumference, height, and waist circumference, it is a better predictor of all-cause mortality than BMI alone.^34^ In kidney transplant recipients, when adjusting for waist circumference (which is significantly associated with increased posttransplant mortality), a higher BMI is paradoxically associated with lower posttransplant mortality.^35^ Therefore, BMI in isolation may not be the ideal metric to gauge risk. Although waist circumference is a valuable measure of obesity, it requires intentional measurement and is not available in most registries. Conversely, BSA can be determined based on patient weight and height; the same variables required to ascertain BMI. As patients with obesity often face barriers to access to transplantation,^36^ substratifying risk profiles based on concordance or discordance with BSA may help better prognosticate risk allowing access to transplant for patients with an elevated BMI, but normal BSA. A BMI-BSA combined approach may be better than BMI alone for identifying patients with ESKD who most benefit from transplantation.

BMI and BSA are both measure of adiposity but reflect different factors. BMI only assesses the relative amount of body fat and tends to overestimate body fat in individuals who are muscular and lean.^37^ Alternatively, BSA measures the surface area of the body and is less affected by body fat and more so by the overall size of an individual^18^; large individuals who are lean may still have a BSA which classifies them as obese. This likely explains the discordance in our data; 9.9% of patients were classified as having an obese BMI and nonobese BSA, whereas 6.5% were classified as having a nonobese BMI and obese BSA. Notably, the BSA of a person who is short and obese will be less than someone who is tall with normal weight.^38^ A person who is tall with normal weight may be expected to have an elevated BSA, but a normal or nonobese BMI. The other discordant situation where BMI is obese and BSA is not (9.9%) may be explained by patients with increased muscle mass and short stature; this may result in an obese BMI, but low BSA.^39,40^ Importantly, irrespective of which metric met threshold for obesity, discordant BMI and BSA (obese BMI-nonobese BSA and nonobese BMI-obese BSA) were associated with an identical risk of DCGL, and no risk for ACGL. These results indicate that there are subgroups of recipients with and without BMI-defined obesity who may be at variable risk of graft loss depending on whether they meet threshold for *BSA*-defined obesity. Although these risks are lower than for patients with combined obese BMI-BSA, these results, like other literature,^41^ suggest that additional patient characteristics can influence outcomes for patients with severe obesity and eligibility should not be determined solely based on BMI thresholds.^42^

This work highlights the established limitations of BMI. These include its overestimation of obesity and inability to differentiate weight that is based on adiposity or muscle.^43^ Furthermore, BMI is unable to differentiate between visceral and subcutaneous abdominal fat distributions,^1,44^ which may have differential renal implications.^1^ BMI-defined obesity has been associated with increased risk of posttransplant complications,^7,45^ which is why many programs limit access to transplant to those with a BMI <40 kg/m^2^.^8^ Even when programs do not incorporate absolute BMI thresholds to establish transplant eligibility, it has been shown that patients with higher BMIs experience longer wait times for transplant,^36,46^ particularly among women.^46,47^ However, transplant literature shows that patients with kidney failure and obesity benefit from KT relative to remaining on dialysis, even with a BMI ≥40 kg/m^2^.^12,17^

The fact that BMI-defined obesity risk can be subcategorized into patients with obese or nonobese BSA, and that there are significant differences in risk profiles for these 2 subcohorts is an important consideration. Although we do not advocate those patients with obesity defined using BMI be limited in access to transplantation, if obesity-related risk is a concern, there may be benefit in further substratifying these patients by corresponding BSA-defined obesity, which we show here to have important implications in terms of both short- and long-term risk. To our knowledge, there are fewer limitations with BSA. Although there are little to no data regarding BSA-related obesity risk amongst transplant patients, one of the limitations of BSA use is that it does not reliably identify adiposity and subsequent risk of metabolic consequences, particularly in large individuals who are lean. Like BMI, BSA also has limitations regarding the assessment of adipose distribution with potential to over or under estimate obesity, especially abnormal body fat, which least influences BSA.^18^ Additionally, both tools only account for height and weight; important metabolic and cardiovascular comorbid conditions including implications of the metabolic syndrome, are not considered in either metric. However, we have shown that an obese BSA is associated with higher risks of DCGL, ACGL, and DGF. BSA is not commonly used to assess obesity; thus, the literature is limited regarding established obesity thresholds and in acknowledging its limitations as a clinical weight parameter tool.

Although our study has several strengths including its novelty in examining the combined exposure of BMI and/or BSA-defined obesity and associations with transplant outcomes, there are important limitations for consideration. First, the retrospective nature of our study poses an inherent risk of miscoding and misclassification of variables. When analyzing our discordant data, the scenario of obese BMI and nonobese BSA (n = 23 922; 9.9%) and nonobese BMI and obese BSA (n = 15 678; 6.5%) was relatively infrequent which limited power to confidently detect risk in this population. However, despite this, although confidence intervals were slightly wider in these discordant cohorts reflecting this increased uncertainty, for the outcomes of DCGL and DGF, confidence intervals did not cross 1 indicating that the true risk was likely increased for those with discordance between BMI and BSA. Given potential programmatic restrictions for patients with increased BMI, it is possible patients with obesity selected for transplant may represent a healthier population of patients (ie, only the healthiest patients with BMI-defined obesity permitted access to transplant); however, if this were the case, it would only bias our results toward the null hypothesis, and the true point estimate of risk may be even higher. In addition, our models were adjusted for comorbidities including diabetes, coronary artery disease, and hypertension. Given the association of these comorbidities with the metabolic syndrome, adjusting for these factors may have also minimized the true risk associated with obesity (defined by either metric), although unadjusted models were similar. Finally, this study was unable to assess whether changes in BMI and/or BSA posttransplant contributed to longer-term outcomes. It has been shown that weight gain is common postkidney transplant with as many as 54.6% of patients experiencing weight gain by 1 y posttransplant^48^; ongoing weight gain posttransplant may have further influenced our findings yet was unable to be accounted for. As such, future studies should track BMI and BSA trends prospectively posttransplant to assess how changes in either may contribute to different transplant outcomes. Nevertheless, we believe combined BMI-BSA may better analyze the risk of patients with obesity compared with either measure alone.

This study demonstrates that combined BMI-BSA-defined obesity is associated with an increased risk of DCGL, ACGL, and DGF relative to patients with nonobese BMI-BSA or discordant BMI-BSA. Combined BMI-BSA-defined obesity should be considered when defining obesity-related risk at the time of kidney transplantation.

## Supplementary Material


