# Supplementary material for: Combined Body Mass Index and Body Surface Area to Predict Post Kidney Transplant Outcomes in Patients With Obesity
Source: Transplant Direct. 2025 May 21;11(6):e1807. doi: 10.1097/TXD.0000000000001807 (PMC12097780; doi:10.1097/TXD.0000000000001807)
Supplement: Supplementary file 1 [file txd-11-e1807-s001.pdf]

Supplement

Table S1. Univariable analysis of weight parameter's effect on death censored graft loss, all cause graft loss, and delayed graft function

	DCGL (HR)	ACGL (HR)	DGF (OR)
Obese BMI- Obese BSA	1.27 (1.24-1.30)	1.20(1.18-1.22)	1.61(1.57-1.65)
Obese BSA	1.22 (1.19-1.24)	1.14(1.13-1.16)	1.43(1.40-1.46)
Obese BMI	1.20(1.18-1.23)	1.18(1.16-1.20)	1.55(1.51-1.58)

Table S2 Multivariable analysis of the effect of BMI-BSA on death censored graft loss, all cause graft loss, and delayed graft function with thresholds for BMI and BSA defined obesity identified using sex-specific restricted cubic splines.

	DCGL (HR)	ACGL (HR)	DGF (OR)
Obese BMI - Obese BSA	1.27 (1.22-1.33)	1.16(1.13-1.20)	1.79(1.70-1.88)
Nonobese BMI - Obese BSA	1.20(1.15-1.27)	1.09(1.05-1.13)	1.34(1.25-1.42)
Obese BMI – Nonobese BSA	1.16(1.11-1.23)	1.05(1.01-1.09)	1.46(1.37-1.54)

**Adjusted for preemptive kidney transplant, previous kidney transplant, cause of end-stage kidney disease, donor and recipient age, race, sex, donor body mass index, panel reactive antibody, human leukocyte antigen mismatch, donor type, and recipient comorbidities (diabetes, peripheral vascular disease, coronary artery disease, hypertension)*

Table S3 Multivariable analysis of the effect of BMI-BSA on death censored graft loss, all cause graft loss with BSA defined using the DuBois formula.

	DCGL (HR)	ACGL (HR)	DGF (OR)
Obese BMI - Obese BSA	1.25 (1.21-1.28)	1.10(1.08-1.12)	1.60(1.54-1.65)
Nonobese BMI - Obese BSA	1.13(1.08-1.18)	1.04(1.01-1.07)	1.19(1.13-1.26)
Obese BMI – Nonobese BSA	1.13(1.09-1.17)	1.02(1.00-1.04)	1.36(1.31-1.41)

**Adjusted for preemptive kidney transplant, previous kidney transplant, cause of end-stage kidney disease, donor and recipient age, race, sex, donor body mass index, panel reactive antibody, human leukocyte antigen mismatch, donor type, and recipient comorbidities (diabetes, peripheral vascular disease, coronary artery disease, hypertension)*

Figure S1. Univariable analysis examining risk of (a) Death-Censored Graft Loss and (b) All Cause Graft Loss based on BMI-BSA pairing

a)

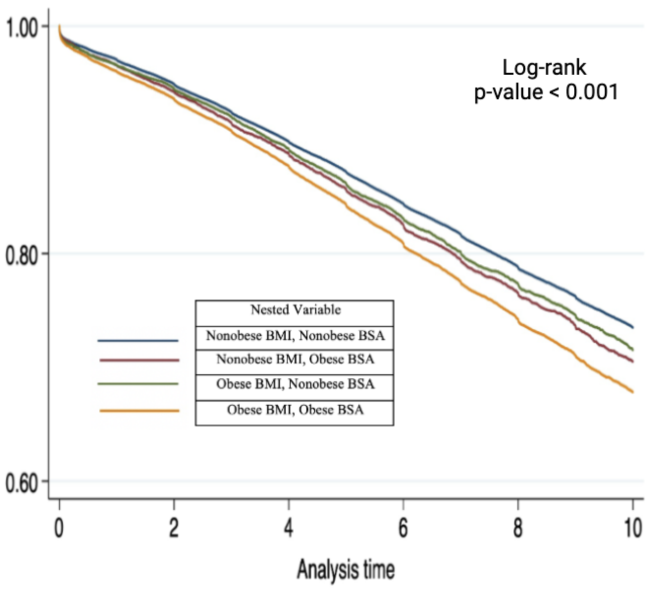

b)

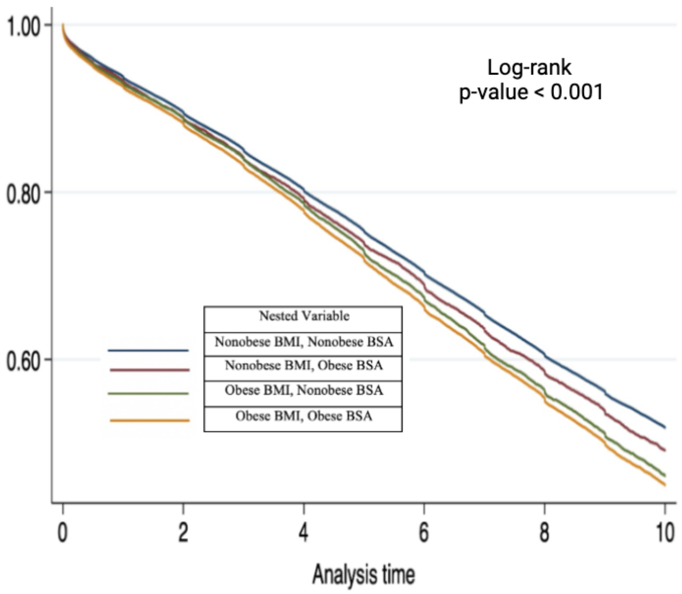

Figure S2. Patient survival based on BMI-BSA pairing for transplant recipients with obesity

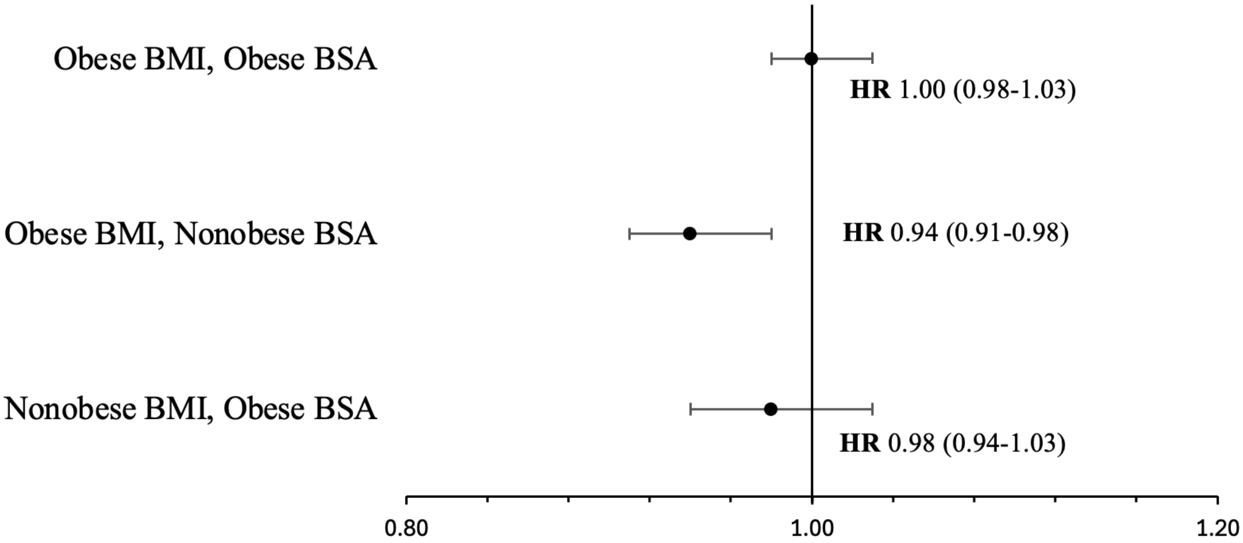

** Adjusted for preemptive kidney transplant, previous kidney transplant, cause of end-stage kidney disease, donor and recipient age, race, sex, donor body mass index, panel reactive antibody, human leukocyte antigen mismatch, donor type, and recipient comorbidities (diabetes, peripheral vascular disease, coronary artery disease, hypertension)*
